# Elite-Guided Nutcracker Optimization Algorithm with Memory-Differential Spiral Search for Feature Selection

**DOI:** 10.3390/biomimetics11100684

**Published:** 2026-09-22

**Authors:** Baowei Pang, Chunying Kang

**Affiliations:** School of Computer and Big Data, Heilongjiang University, Harbin 150080, China; 2242775@s.hlju.edu.cn

**Keywords:** feature selection, multidimensional datasets, nutcracker optimization algorithm, mRMR, logistic–tent chaotic mapping, memory-differential spiral search, elite-guided search

## Abstract

Feature selection can effectively remove redundant and irrelevant features in classification tasks; however, feature subset search on datasets with different dimensionalities still faces challenges such as strong initialization randomness, complex search spaces, a single local exploitation direction, and difficulty in effectively updating inferior individuals. This paper proposes an Elite-Guided Nutcracker Optimization Algorithm with Memory-Differential Spiral Search for Feature Selection (EGRNOA). The proposed method first employs Minimum Redundancy Maximum Relevance (mRMR) to preselect candidate features, thereby reducing the interference of redundant features in the subsequent search process. Then, Logistic–Tent chaotic initialization is used to enhance the uniformity of the initial population distribution. In the foraging phase of NOA, a memory-differential spiral search strategy is designed in which global best memory, individual historical best memory, and random differential directions are jointly incorporated into the spiral updating process so that the search no longer contracts only around a single optimal individual, thereby enhancing local exploitation capability while maintaining directional perturbation. A diversity-adaptive elite-guided reconstruction mechanism for inferior individuals is constructed; this mechanism uses elite population information to directionally repair inferior individuals, which improves population update efficiency while alleviating premature convergence. Experimental results on the CEC2020 benchmark functions show that EGRNOA achieves the best or tied-best average results on all ten functions and obtains the best Friedman average ranking of 1.050. On ten classification datasets with different dimensionalities, EGRNOA achieves the highest average classification accuracy on nine; across datasets, its average accuracy on the movement dataset is improved by 6.41 percentage points when compared with the second-best algorithm. Convergence curves, box plots, critical difference diagrams, and ablation experiments further verify the effectiveness of the memory-differential spiral search and diversity-adaptive elite-guided reconstruction mechanism in improving search quality and enhancing the robustness of feature selection.

## 1. Introduction

With the rapid development of data acquisition and intelligent sensing technologies, classification data in fields such as medical diagnosis, bioinformatics, text analysis, image recognition, cybersecurity, and industrial fault diagnosis exhibit different dimensional characteristics. Some datasets contain only a small number of descriptive variables, whereas others include hundreds or even thousands of candidate features. Regardless of the dimensional scale, the original feature space may contain irrelevant features, redundant features, weakly relevant features, and noisy variables. Directly using all original variables not only increases training costs but may also reduce the generalization ability and stability of the model [1,2,3,4]. Therefore, selecting a compact and discriminative feature subset is an important problem in machine learning, pattern recognition, and biomedical data analysis [5,6,7,8].

Feature selection aims to remove unnecessary information from the original feature set while retaining variables that contribute to the target task [1,2,3,9]. Unlike feature extraction, feature selection does not alter the physical meaning of the selected variables; this confers advantages in scenarios that emphasize interpretability, including medicine, biology, industrial monitoring, and security analysis. Existing methods are generally divided into filter, wrapper, and embedded methods: filter methods are computationally efficient, but are weakly coupled with the final classifier; wrapper methods directly evaluate candidate subsets using a classifier and usually achieve better predictive performance, however, repeated classifier evaluations increase the computational cost [10,11]; and embedded methods integrate feature selection into the model training process, such as importance evaluation based on tree models, but their results often depend on the specific learning model [12].

The challenges of feature selection do not exist only in high-dimensional data; although the search space in low-dimensional datasets is relatively smaller, an unstable optimizer may still mistakenly remove key variables or cause overfitting on limited samples. In medium-dimensional datasets, the interactions among variables become more complex and redundant information begins to affect the quality of feature subsets. In high-dimensional datasets, the feature subset space grows exponentially, making exhaustive search infeasible and imposing higher requirements on initialization quality, population diversity maintenance, and local refinement capability [9,13,14]. Therefore, instead of being designed only for a specific dimensional condition, practical feature selection algorithms should be able to maintain stable performance across datasets with different dimensional scales.

Early evolutionary computation and swarm intelligence algorithms, including genetic algorithms, particle swarm optimization, and ant colony optimization, can provide fundamental frameworks for feature subset search [15,16,17]. Metaheuristic algorithms, including Grey Wolf optimization, whale optimization, the sine cosine algorithm, and Harris Hawk optimization, have also been widely applied to complex optimization problems [18,19,20,21]. These algorithms do not rely on gradient information and can maintain multiple candidate solutions during the search process, making them suitable for handling discrete, nonlinear, and multimodal feature subset optimization problems. However, their performance is still affected by population initialization, the balance between exploration and exploitation, diversity maintenance, boundary handling, and the strategy for mapping from continuous positions to binary feature subsets [22,23,24,25].

The Nutcracker Optimization Algorithm (NOA) is a swarm intelligence optimization method that simulates the foraging, caching, spatial memory, and cache recovery behaviors of nutcrackers [26,27,28]. In NOA, individual positions represent candidate solutions, and the population is alternately updated in the search space through two types of behaviors: foraging and caching, and cache searching and recovery. The former focuses on searching for and storing potentially promising positions, whereas the latter re-searches cached regions by using reference points and historical memory, thereby forming a dynamic balance between exploring unknown regions and exploiting advantageous regions. In recent years, NOA and its improved variants have been applied to tasks such as association rule mining, antenna design, engineering optimization, camera calibration, and path planning, demonstrating promising extensibility [29,30,31,32,33].

To address the above problems, this paper proposes an improved NOA-based feature selection method for feature datasets with different dimensionalities. First, mRMR is adopted as a flexible coarse-grained screening strategy to compress candidate variables when the original feature space is large or highly redundant, while still retaining task-relevant attributes for subsequent search [34,35]. Second, since related studies have shown that chaotic mechanisms can be used to enhance the search diversity of swarm intelligence optimizers [36], Logistic–Tent chaotic initialization is introduced to improve the coverage of the initial population. Third, a memory-differential spiral search strategy is proposed in the foraging phase of NOA; with this strategy, the spiral updating process no longer performs single-anchor contraction only around the current global best position, instead simultaneously utilizing global best memory, individual historical best memory, and random differential foraging directions. Fourth, a diversity-adaptive elite-guided reconstruction mechanism is designed to reconstruct inferior individuals through elite guidance, dual-mode updating, reflective boundary handling, and a probabilistic acceptance criterion. Finally, sigmoid binary mapping and KNN with five-fold cross-validation are combined to evaluate candidate feature subsets [24,37,38,39].

The main contributions of this paper are summarized as follows. (1) An mRMR-assisted candidate feature screening strategy is employed to reduce redundant search dimensions while enabling the proposed method to adapt to datasets with different feature scales. (2) A Logistic–Tent chaotic initialization method is used to improve population diversity and initial coverage. In the foraging phase of NOA, a memory-differential spiral search strategy transforms the traditional single-anchor spiral update into a search mode jointly driven by global best memory, individual historical best memory, and random differential foraging directions, thereby enhancing local exploitation capability and reducing the risk of individuals prematurely aggregating toward the current best position. (3) A diversity-adaptive elite-guided reconstruction mechanism uses elite population information to directionally reconstruct inferior individuals, thereby improving the quality of population updates in the later stage and alleviating premature convergence. (4) Finally, the above mechanisms are combined with binary mapping and KNN evaluation to form a unified metaheuristic feature selection framework. The remainder of this paper is organized as follows: Section 2 reviews the related work; Section 3 presents the proposed EGRNOA-based feature selection method; Section 4 reports and discusses the experimental results; and Section 5 concludes the paper.

## 2. Related Work

This section reviews two aspects related to the proposed EGRNOA framework: feature screening and dimensionality reduction for datasets with different feature scales, and feature subset optimization methods based on metaheuristic algorithms.

### 2.1. Feature Screening and Dimensionality Reduction

Classification data do not exhibit a single dimensional form: low-dimensional data may contain only a small number of candidate variables, but can still be affected by noise and limited sample sizes; medium-dimensional data often include correlated variables and feature interactions that are difficult to evaluate independently; and high-dimensional data further amplify redundancy, irrelevant information, and computational burden. Therefore, feature screening should not be regarded as a technique used only in extremely high-dimensional scenarios but instead as a general mechanism for controlling the quality of the candidate feature space [1,2,3,4].

Filter-based screening methods do not require repeated classifier training, as they evaluate features according to statistical or information-theoretic criteria. Common criteria include correlation coefficients, chi-square statistics, relief-based measures, mutual information, symmetrical uncertainty, and minimum redundancy–maximum relevance (mRMR) [34,35,40,41]. Such methods are computationally efficient and can rapidly remove obviously weak variables; however, because the evaluation process is usually independent of the final classifier, purely filter-based methods may ignore feature interactions or remove variables that become valuable only when combined with other attributes.

Feature ranking and candidate preselection methods can establish a practical connection between independent feature evaluation and subsequent subset optimization. By ranking variables according to relevance and redundancy criteria, the optimizer can search in a cleaner candidate space without explicitly dividing features into several groups. This strategy is meaningful across different dimensional scales. In smaller datasets, it reduces the risk of mistakenly removing effective features by retaining task-relevant candidate variables. In larger or highly redundant datasets, it can reduce unnecessary search burden before classifier-based subset evaluation [5,6,9,14].

### 2.2. Feature Selection Methods Based on Metaheuristic Algorithms

Feature selection is essentially a combinatorial optimization problem. For a dataset containing *D* original features, the number of possible feature subsets is $2^{D}$. Although this search space may still be manageable in extremely small-scale cases, exhaustive search quickly becomes infeasible as *D* increases. According to the No Free Lunch theorem, no optimizer can consistently outperform all other methods across all problems; therefore, it is necessary to adapt optimization strategies to the structure of specific problems [13]. Metaheuristic algorithms have been widely used for feature selection tasks because they do not rely on gradient information, have flexible structures, and can explore multiple subspace regions through population-based search [42,43,44,45].

Classical evolutionary algorithms and swarm intelligence algorithms such as genetic algorithms, particle swarm optimization, ant colony optimization, Grey Wolf optimization, and whale optimization have been applied to feature selection through different encoding schemes and fitness functions. In these methods, a candidate solution usually represents a feature subset, while the fitness function is constructed based on classification accuracy, classification error, feature selection ratio, or their weighted combination. Compared with simple independent feature ranking, these algorithms can more easily characterize interactions among features, making them particularly suitable for cases where the effect of a variable depends on whether other variables are selected [11,23,24,25].

Nevertheless, metaheuristic feature selection still faces common challenges across different dimensional scales: for low-dimensional data, the main risk lies in unstable subset selection caused by limited samples or weak signal differences; for medium-dimensional data, redundancy and variable interactions increase the complexity of the fitness landscape; and for high-dimensional data, the search space expands rapidly and the cost of repeated wrapper-based evaluations becomes higher. Therefore, recent studies have increasingly focused on enhancing the internal search mechanisms of optimizers, including improved initialization, adaptive exploration and exploitation, diversity maintenance, and local refinement capability [22,36,42,43].

## 3. Design of the Improved NOA-Based Feature Selection Algorithm

Although the standard NOA has a certain degree of population-based cooperative search capability, its original model is mainly designed for continuous optimization problems. When directly applied to feature selection, NOA not only needs to address the mapping problem from continuous positions to binary feature subsets but must also deal with issues such as candidate feature redundancy, insufficient initial population coverage, a single local exploitation direction in the foraging phase, and low update efficiency of inferior individuals in the later stage. These problems jointly affect the search stability of the algorithm and the quality of the selected feature subsets across datasets with different dimensionalities.

To address these problems, this paper constructs the EGRNOA framework for feature selection. This framework is developed around the main objectives of reducing search space complexity, enhancing the exploitation capability in the foraging phase, and improving the update quality of inferior individuals. First, mRMR is used to perform necessary compression of the candidate feature space. Then, Logistic–Tent initialization is adopted to improve the population distribution. In the foraging phase of NOA, memory-differential spiral search is embedded so that the spiral update is simultaneously constrained by global memory, individual memory, and differential directions. Furthermore, a diversity-adaptive elite-guided mechanism is combined to reconstruct inferior individuals. Finally, feature subset evaluation is completed through sigmoid binary mapping and KNN cross-validation.

### 3.1. Overall Framework of the Improved Algorithm

The proposed improved NOA-based feature selection algorithm generally consists of two layers: the first is a coarse-grained preselection layer based on the filter strategy, while the second is a fine-grained NOA search layer based on the wrapper strategy. The former is used to rapidly remove a large number of irrelevant features and some redundant features, whereas the latter further searches for better feature subsets in the compressed candidate feature space.

The specific procedure is as follows. The original dataset is input and the mRMR method is used to rank the original features, with candidate features selected according to a preset retention ratio to form a coarsely screened feature subspace. Within this candidate subspace, Logistic–Tent chaotic mapping is used to generate the initial population, thereby enhancing the distribution uniformity of individuals; iterative updating is then performed under the main search framework of the standard NOA. In this process, the memory-differential spiral search strategy is introduced in the foraging phase to enhance local neighborhood exploitation capability. In the middle and later stages, the diversity-adaptive elite-guided reconstruction mechanism is triggered according to population diversity and individual quality to directionally optimize inferior individuals. Next, the updated continuous individuals are mapped into binary feature vectors and the classification accuracy is calculated using a KNN classifier. The fitness function is constructed by combining classification accuracy with the feature compression ratio. Finally, the optimal feature subset and the corresponding classification performance metrics are output.

Therefore, the proposed method can be summarized as a unified solution framework integrating mRMR-based coarse screening, chaotic initialization, memory-differential spiral search-enhanced foraging, elite-guided reconstruction of inferior individuals, and binary discretization evaluation. Compared with the standard NOA, the proposed method no longer relies solely on random initialization and conventional updating strategies, now systematically improving search space compression, search capability enhancement, and population quality improvement for the feature selection problem.

### 3.2. Initial Feature Preselection Strategy Based on mRMR

#### 3.2.1. Design Motivation

In feature selection tasks, the original data often contain a large number of irrelevant and redundant features. If all features are directly input to a wrapper-based optimization algorithm for search, then the search space will be significantly enlarged and the number of classifier evaluations will increase, reducing overall search efficiency. In particular, when the original dimensionality is high, the optimization algorithm will need to repeatedly search among a large number of low-quality features, which can easily lead to slow convergence and stagnation in local optima.

To reduce the complexity of the search space, this paper introduces a coarse-grained feature preselection strategy based on Minimum Redundancy–Maximum Relevance (mRMR) before executing the improved NOA. The core idea of this strategy is to retain features that are strongly relevant to the class labels while minimizing the redundancy among the retained features, thereby providing a more compact candidate feature space for subsequent wrapper-based search.

#### 3.2.2. Principle of mRMR-Based Preselection

Let the original feature set be $F=\{f_{1},f_{2},\ldots ,f_{D}\}$, the class label be *c*, and the selected feature subset be *S*. Then, mRMR mainly evaluates candidate features from the following two aspects.

1. Maximum Relevance:

(1) $$D\left(f_{j}\right)=I\left(f_{j};c\right)$$

where $I(f_{j};c)$ denotes the mutual information between feature $f_{j}$ and the class label c; a larger value indicates that the feature is more helpful for class discrimination.

2. Minimum Redundancy:

(2) $$R\left(f_{j}\right)=\frac{1}{\left|S\right|}\sum_{f_{k}\in S}I\left(f_{j};f_{k}\right)$$

where $I(f_{j};f_{k})$ denotes the mutual information between the candidate feature and the selected features; a smaller value indicates that less redundant information exists between the candidate feature and the selected features.

By combining these two terms, the evaluation criterion of mRMR can be obtained as follows:

(3) $$\mathrm{Score}\left(f_{j}\right)=D\left(f_{j}\right)-R\left(f_{j}\right)$$

that is, features are preferentially selected if they have high relevance to the class labels and low redundancy with the selected features.

#### 3.2.3. Design of the Coarse Screening Rule

To ensure that the preselection strategy can compress the search space while avoiding excessive feature removal that may lead to information loss, this paper designs a segmented retention rule according to the original feature dimensionality:

(1) When the original dimensionality is less than 300, no coarse screening is performed and the algorithm directly enters the fine-grained search phase of the improved NOA.
(2) When the original dimensionality is between 300 and 1000, the top 60% of features are retained.
(3) When the original dimensionality is greater than 1000, the top 50% of features are retained.

Let the original feature dimensionality be *D* and the number of retained features after coarse screening be *K*. Then, the candidate feature retention ratio can be expressed as follows: 

(4) $$R_{\mathrm{pre}}=\frac{K}{D}.$$

Through this segmented strategy, the proposed method controls the scale of the search space while maintaining the ability to retain useful information across datasets with different dimensionalities.

It should be noted that mRMR-based preselection is used only to obtain the candidate feature index set. During the subsequent EGRNOA search process, each binary individual represents a feature subset selected from this candidate index set. During fitness evaluation, the algorithm extracts the corresponding feature columns from the original dataset according to the indices encoded by the selected subset, then computes the KNN classification accuracy using five-fold cross-validation. Therefore, the classification evaluation is still performed on the actual features in the original data space, with the role of mRMR limited to compressing the candidate search space.

### 3.3. Multi-Strategy Improved Nutcracker Optimization Algorithm

#### 3.3.1. Initialization Strategy Based on Logistic–Tent Chaotic Mapping

In metaheuristic optimization algorithms, the initialization phase determines the initial coverage of the population in the search space. If the initial individuals are unevenly distributed, some regions may be oversampled while others cannot be sufficiently explored, thereby affecting the subsequent search performance. The standard NOA usually adopts random initialization, which is simple to implement but may suffer from individual aggregation and unstable distribution quality in feature selection tasks.

To improve the initial distribution quality of the population, this paper uses Logistic–Tent chaotic mapping to generate the initial individuals. Since chaotic sequences have good ergodicity, sensitivity to initial values, and pseudo-randomness, they can produce a more uniform distribution of individuals during the initialization phase.

Let the chaotic variable be $x_{k}$. Then, the Logistic–Tent hybrid mapping adopted in this paper can be expressed as follows:

(5) $$x_{k+1}=\left\{\begin{array}{cc} \mathrm{mod}\left(rx_{k}\left(1-x_{k}\right)+\frac{\left(4-r\right)x_{k}}{2},\;1\right),\; & x_{k}<0.5,\\ \mathrm{mod}\left(rx_{k}\left(1-x_{k}\right)+\frac{\left(4-r\right)\left(1-x_{k}\right)}{2},\;1\right), & x_{k}\geq 0.5, \end{array}\right.$$

where *r* is the control parameter and mod(1) is used to restrict the mapping result to the interval [0,1]. This mapping integrates the nonlinear characteristics of the logistic map and the uniform traversal property of the tent map, and can be used to generate a more stable initialization sequence.

After generating an N×dim chaotic matrix, it is mapped to the actual search space as follows:

(6) $$X_{ij}=lb_{j}+c_{ij}\left(ub_{j}-lb_{j}\right)$$

where $c_{ij}$ denotes an element of the chaotic sequence, with $ub_{j}$ and $lb_{j}$ as the upper and lower search bounds of the *j*-th dimension, respectively.

#### 3.3.2. Memory-Differential Spiral Search Strategy

In the foraging phase of the standard NOA, individuals mainly update their positions based on the guidance of the current best solution, differential information, and random perturbation. If traditional WOA-style spiral search is directly adopted, individuals usually contract only around the current global best position. Although this strategy can enhance local exploitation, it may also cause multiple individuals to prematurely aggregate in the current best region, which weakens the original random differential search capability of the NOA foraging phase and reduces the diversity of subsequent search directions. Therefore, it is necessary to specifically modify the spiral search according to the foraging behavior of NOA and the requirements of feature selection tasks.

To prevent spiral search from becoming a simple single-anchor contraction process, this paper proposes a memory-differential spiral search strategy. In this strategy, the spiral center is extended from a single global best position to a memory anchor jointly constructed using the global best position and the individual historical best position. Meanwhile, the random differential direction already present in the NOA foraging phase is introduced to correct the spiral trajectory. In this way, individuals can still retain their own historical memory and population differential perturbation information while they approach the current promising region, which improves both the stability of local exploitation and the diversity of search directions in the foraging phase.

Let the position of the *i*-th individual in the *j*-th dimension at the *t*-th iteration be $X_{i,j}^{t}$, the current global best position be $X_{\mathrm{best},j}^{t}$, the individual historical best position be $L_{i,j}^{t}$, and two randomly selected individuals be $X_{cv,j}^{t}$ and $X_{cv1,j}^{t}$. Then, the memory anchor, differential direction, and spiral updating method are respectively defined as follows:

(7) $$M_{i,j}^{t}=X_{\mathrm{best},j}^{t}+\omega_{m}^{t}r_{1}\left(L_{i,j}^{t}-X_{\mathrm{best},j}^{t}\right)$$

(8) $$\Delta_{i,j}^{t}=\omega_{d}^{t}r_{2}\left(X_{cv,j}^{t}-X_{cv1,j}^{t}\right)$$

(9) $$X_{i,j}^{t+1}=M_{i,j}^{t}+\left|M_{i,j}^{t}-X_{i,j}^{t}\right|\exp\left(q\right)\cos\left(2\pi q\right)+\Delta_{i,j}^{t}$$

where $M_{i,j}^{t}$ denotes the memory anchor, $\Delta_{i,j}^{t}$ denotes the differential foraging direction, $\omega_{m}^{t}$ and $\omega_{d}^{t}$ denote the adaptive decay weights of the memory term and the differential term, respectively, with $\omega_{d}^{t}=0.3\left(1-\frac{t}{T}\right)$ and $\omega_{m}^{t}=0.6\left(1-\frac{t}{T}\right)$, and *q* is a random variable uniformly distributed in the interval [−1,1], used to control the rotation direction and perturbation amplitude of the spiral trajectory. As the iteration proceeds, the memory term and the differential term gradually weaken, allowing the algorithm to maintain strong search tension in the early stage and gradually shift toward fine-grained exploitation near the current promising region in the later stage. Compared with traditional single-anchor spiral search, this strategy is more consistent with the search characteristics of the NOA foraging phase, which simultaneously utilizes optimal memory, individual memory, and random differential directions.

#### 3.3.3. Diversity-Adaptive Elite-Guided Reconstruction Mechanism

As iteration proceeds, individuals in the NOA population gradually aggregate toward the current promising region. Although such aggregation facilitates later-stage convergence, it also leads to a decline in population diversity. This makes it difficult for individuals with poor fitness to escape from low-quality regions, weakening the overall activity of the population. In feature selection tasks in particular, if inferior individuals remain near low-quality solutions for a long time, then the risk of the algorithm falling into local optima increases.

To address this problem, this paper proposes a diversity-adaptive elite-guided reconstruction mechanism. This mechanism does not simply reinforce the current best individual; instead, it performs targeted reconstruction of inferior individuals according to the population diversity level and fitness ranking results, which improves the search quality of the entire population.

Population diversity is the quantitative description of the distribution state of the population. In this paper, population diversity is formally defined as follows:

(10) $$\mathrm{Diversity}=\frac{1}{\dim}\sum_{d=1}^{\dim}\frac{\mathrm{std}\left(x_{d}\right)}{\max\left(ub_{d}-lb_{d},\varepsilon \right)}$$

where $std\left(x_{d}\right)$ denotes the standard deviation of the population in the *d*-th dimension, $ub_{d}$ and $lb_{d}$ denote the upper and lower bounds of this dimension, respectively, and ε is a small constant used to prevent the denominator from being zero. A smaller value of this indicator indicates that the population is more concentrated and that the risk of premature convergence is higher.

For elite subgroup construction and inferior individuals, after the population is sorted according to fitness from best to worst, let the top individuals constitute the elite subgroup. Then, the mean position of the elite subgroup is defined as follows:

(11) $$E=\frac{1}{N_{e}}\sum_{i=1}^{N_{e}}X_{i}$$

where *E* denotes the elite mean vector. Unlike guidance, which uses only a single best solution, the elite mean integrates information from multiple promising individuals, which can reduce directional bias caused by an accidentally optimal individual.

Meanwhile, several individuals ranked lower according to fitness are defined as inferior individuals; their intervention ratio is adaptively controlled by population diversity, as follows:

(12) $$R_{\inf}=0.15+0.25\left(1-\mathrm{Diversity}\right).$$

When population diversity decreases, the proportion of inferior individuals requiring intervention increases, thereby strengthening the reconstruction of individuals located in low-quality regions.

An adaptive triggering mechanism is designed to ensure that this mechanism mainly takes effect in the middle and later stages. The triggering probability is related to the iteration process as follows:

(13) $$P_{\mathrm{trig}}=0.1+0.5{\left(\frac{t}{T}\right)}^{1.5}$$

where *t* denotes the current iteration number and *T* denotes the maximum number of iterations. This strategy enables the algorithm to maintain a relatively low intervention frequency in the early stage, ensuring sufficient execution of the basic search process. In the middle and later stages, the triggering probability is gradually increased to alleviate the decline in population diversity and stagnation in local optima.

To meet the requirements of different search stages, this paper designs two update modes for inferior individuals:

1. Escape exploration mode.

When the population diversity is relatively high and the algorithm is still in the early-to-middle stages, the following escape exploration mode is adopted:

(14) $$X_{i}^{\mathrm{new}}=X_{i}+0.6r_{1}⊙\left(E-X_{i}\right)+0.3r_{2}⊙\left(X_{i}-X_{k}\right)+\sigma N\left(0,1\right)⊙\left(ub-lb\right)$$

where $X_{i}$ denotes the inferior individuals, $X_{k}$ denotes a randomly selected reference individual, and σ denotes the perturbation intensity:

(15) $$\sigma =0.1\left(1-\frac{t}{\mathrm{Max}\text{\_}\mathrm{iter}}\right)+0.01.$$

This mode enables inferior individuals to escape from the current low-quality regions through the combined effects of elite guidance, the differential term, and random perturbation.

2. Elite Encircling Exploitation Mode.

When the algorithm enters the later stage or when population diversity decreases significantly, the elite encircling exploitation mode is adopted:

(16) $$X_{i}^{\mathrm{new}}=X_{i}+0.7r_{3}⊙\left(X_{\mathrm{best}}-X_{i}\right)+0.2r_{4}⊙\left(E-X_{i}\right)+0.1r_{5}⊙\left(L_{i}-X_{i}\right)$$

where $X_{\mathrm{best}}$ denotes the current global best position and $L_{i}$ denotes the historical local best position of individual *i*. This mode comprehensively utilizes global best information, elite population information, and individual historical experience to guide inferior individuals to contract toward high-quality regions.

For probabilistic acceptance criterion and boundary control, in the candidate solution acceptance stage, this paper adopts a hybrid criterion consisting of greedy updating plus probabilistic acceptance. If the candidate solution is better than the current solution, it is directly accepted; if the candidate solution is slightly worse, then it is accepted with a certain probability, formulated as follows:

(17) $$P_{\mathrm{acc}}=\exp\left(-\frac{\max\left(f\left(X_{i}^{\mathrm{new}}\right)-f\left(X_{i}\right),0\right)}{\mathrm{Temp}}\right)$$

where Temp denotes the temperature parameter:

(18) $$\mathrm{Temp}=0.05+0.2\left(1-\frac{t}{\mathrm{Max}\text{\_}\mathrm{iter}}\right).$$

This strategy allows a small number of suboptimal solutions to enter the population while maintaining the overall optimization direction, reducing the risk of premature convergence along a single search path.

To prevent updated individuals from exceeding the boundaries, this paper additionally adopts a reflective boundary control strategy to correct new solutions, thereby preserving directional information while ensuring that individuals always remain within the feasible domain.

### 3.4. Binary Discretization Feature Selection Framework and Fitness Evaluation

#### 3.4.1. Mapping from Continuous Solutions to Binary Solutions

NOA is a continuous optimization algorithm in which individual positions are represented as real-valued vectors; however, feature selection requires the output of a binary feature subset. Therefore, it is necessary to establish a mapping relationship between the continuous position vector and the binary selection vector.

Let the individual position be

(19) $$X_{i}=\left[x_{i1},x_{i2},\ldots ,x_{iD}\right];$$

the Sigmoid function is first used to map the continuous position to the probability of selecting a feature:

(20) $$p_{ij}=\frac{1}{1+e^{-2x_{ij}}}$$

where $p_{ij}$ denotes the feature selection probability corresponding to the *j*-th dimension of the *i*-th individual. Then, it is converted into a binary feature vector according to a random threshold mechanism:

(21) $$s_{ij}=\left\{\begin{array}{cc} 1, & \mathrm{rand}<p_{ij},\\ 0, & \mathrm{rand}\geq p_{ij}. \end{array}\right.$$

In this way, the continuous individual position can be mapped into a specific feature subset.

#### 3.4.2. Classification Evaluation and Fitness Function

For each binary feature subset, this paper uses the KNN classifier for performance evaluation and obtains the classification accuracy through five-fold cross-validation. Let the classification accuracy corresponding to feature subset *S* be Acc(S), the total dimensionality of the original features be *D*, and the number of currently selected features be |S|. Then, the fitness function is defined as follows:

(22) $$\mathrm{Fitness}\left(S\right)=\alpha \left(1-\mathrm{Acc}(S)\right)+\beta \frac{\left|S\right|}{D}$$

where α and β denote the weights of the classification error term and the feature proportion term, respectively. In this paper, α=0.6 and β=0.4, indicating that feature compression is considered while ensuring classification performance. The fitness function is formulated as a single-objective minimization problem; therefore, the objective of the algorithm is to minimize the classification error while reducing the number of selected features as much as possible.

### 3.5. Algorithm Procedure and Time–Space Complexity Analysis

#### 3.5.1. Algorithm Procedure

The algorithm workflow is shown in Algorithm 1.
**Algorithm 1** EGRNOA for feature selection**Input:** Original dataset *D*, population size *N*, and maximum number of iterations *T*.
**Output:** Best feature subset $S_{\mathrm{best}}$.
  1:Apply mRMR pre-screening according to the dataset dimensionality and obtain the candidate feature space.  2:Initialize the population *X* using the Logistic–Tent chaotic map.  3:Convert continuous individuals into binary feature subsets and evaluate fitness using KNN cross-validation.  4:Initialize the personal best solutions and the global best solution $S_{\mathrm{best}}$.  5:**while** t<T **do**  6:      Update individuals according to the standard NOA search framework.  7:      Apply memory-differential spiral search in the foraging stage.  8:      Calculate population diversity and determine whether elite-guided reconstruction is triggered.  9:      **if** the trigger condition is satisfied **then**10:            Construct the elite subset and select inferior individuals.11:            Update inferior individuals using diversity-adaptive elite-guided reconstruction mechanism.12:      **end if**13:      Apply reflection boundary handling, probabilistic acceptance, and binary mapping.14:      Re-evaluate fitness and update personal and global best solutions.15:**end while**16:Recover the final feature indices and return $S_{\mathrm{best}}$.


#### 3.5.2. Time Complexity Analysis

Let the population size be *N*, the feature dimensionality be *D*, the dimensionality after coarse screening be *K*, the maximum number of iterations be *T*, and the classifier evaluation complexity be denoted as $O(C_{\mathrm{cls}})$. The main time costs of the algorithm include the following components: (1) the complexity of mRMR-based coarse screening; (2) the complexity of chaotic initialization; (3) the individual update complexity of memory-differential spiral search and elite-guided search; (4) the complexity of binary discretization mapping; and (5) the complexity of KNN-based classification evaluation.

Since the main computational cost in wrapper-based feature selection is usually dominated by classifier evaluation, operations such as Logistic–Tent initialization, memory-differential spiral search, elite-guided reconstruction, and binary mapping mainly involve individual position updating and mapping calculations, for which the additional overhead is relatively small. Therefore, the complexity of a single iteration can be approximately expressed as $O(NK+NC_{\mathrm{cls}})$, and the overall complexity of the algorithm is approximately $O(T\left(NK+NC_{\mathrm{cls}}\right))$. Since K<D, the mRMR-based coarse screening stage can compress the subsequent wrapper-based search space to a certain extent, helping to reduce the overall search cost.

## 4. Experimental Results and Discussion

To verify the effectiveness and robustness of the proposed EGRNOA in continuous optimization and feature selection tasks, two groups of simulation experiments are designed in this section. The first group adopts the CEC2020 benchmark functions to evaluate the general optimization capability of the improved search mechanisms in terms of optimization accuracy, repeated-run stability, and statistical ranking performance. The second group selects ten representative classification datasets with different feature dimensionalities to evaluate the feature selection performance of EGRNOA from the perspectives of classification accuracy and operational stability. The selected comparison algorithms include six representative metaheuristic feature selection or optimization methods: particle swarm optimization (PSO), ant colony optimization (ACO), genetic algorithm (GA), the standard Nutcracker Optimization Algorithm (NOA), hybrid genetic algorithm-based feature selection method (HGAFS), and hybrid particle swarm feature selection method with local search (HPSO-LS-FS). This paper selects PSO, ACO, and GA as representative classical swarm intelligence and evolutionary algorithms in order to provide a comparative basis for traditional metaheuristic search methods. The standard NOA is selected as the direct baseline in order to examine the effectiveness of the proposed improvement strategies compared with the original Nutcracker Optimization Algorithm. HGAFS and HPSO-LS-FS are selected as representative hybrid feature selection methods for comparison with existing improved feature selection frameworks. These baselines cover classical evolutionary algorithms, swarm intelligence algorithms, the original NOA, and hybrid feature selection methods, enabling a relatively comprehensive evaluation of the relative performance of EGRNOA in feature selection tasks. To ensure the objectivity of the evaluation results and reduce the influence of data partitioning on classification performance, all classification experiments strictly follow a standard five-fold cross-validation procedure.

### 4.1. Experimental Settings and Evaluation Metrics

#### 4.1.1. Benchmark Functions, Datasets, and Classifier

The experiments consist of two parts: the first part adopts the CEC2020 benchmark functions to verify the performance of the improved search mechanism on continuous optimization problems [46], while the second part uses ten classification datasets with different dimensionalities to evaluate the feature selection capability of the algorithm. The dataset sources and machine learning implementation follow the settings of public UCI datasets and commonly used experimental tools [39,47], Table 1 provides a detailed list of the dataset contents. In the classification tasks, candidate feature subsets are evaluated using a KNN classifier combined with five-fold cross-validation [37,38].

#### 4.1.2. General Experimental Parameter Settings

Regarding the experimental parameters settings, to ensure fairness and comparability, this section follows the principle of combining global consistency with local specificity. First, for all comparative swarm intelligence algorithms involved in the classification datasets, the initial population size *N* is uniformly set to 20 and the maximum number of iterations to 100. In order to eliminate the random effects caused by random initialization, all simulation experiments are independently executed 20 times under the same software and hardware environment. The final statistical results are reported as average values, and classification performance is mainly evaluated using accuracy.

#### 4.1.3. Parameter Sensitivity Analysis

Figure 1 presents the changes in the feature selection rate and classification accuracy under different mRMR feature retention ratios. It can be observed that as the mRMR retention ratio gradually increases from a relatively low level, the feature selection rate on the four datasets shows an overall upward trend. This indicates that a higher candidate feature retention ratio expands the subsequent search space of EGRNOA and increases the number of finally selected features. However, the classification accuracy does not continuously improve as the retention ratio increases: on the arcene, parkinson, and isolet datasets, medium retention ratios achieve relatively good accuracy; on the CNAE dataset, a higher retention ratio yields slightly higher accuracy but also results in a higher feature selection rate. Considering both classification accuracy and feature compression performance, this paper adopts (0.5,0.6) as the mRMR retention ratio.

Figure 2 show the changes in the feature selection rate and classification accuracy under different combinations of fitness function weights. It can be observed that when the accuracy weight α increases and the feature number penalty weight β decreases, the overall feature selection rate increases; this indicates that the algorithm tends to retain more features in order to improve classification performance. In terms of classification accuracy, α=0.6 and β=0.4 achieve relatively stable results on most datasets. Although further increasing α may provide slight accuracy improvements on individual datasets, it usually increases the final number of selected features and weakens the compression effect of feature selection. Therefore, this paper adopts α=0.6 and β=0.4 as the default weight setting in order to achieve a relatively balanced trade-off between classification accuracy and feature subset size.

### 4.2. General Optimization Performance Evaluation

EGRNOA serves as the core optimization engine in the proposed feature selection framework. To verify its robustness and generalization capability on continuous numerical optimization problems, this paper adopts the CEC2020 benchmark functions for general optimization performance evaluation. EGRNOA is compared with PSO, ACO, GA, the standard NOA, HGAFS, and HPSO-LS-FS. In the CEC2020 experiments, the population size is set to 20, the maximum number of function evaluations to 200,000, and the problem dimensionality to 20. For each benchmark function, each algorithm is independently run 20 times and the Best, Worst, Mean, and Std values are recorded. Since a lower objective function value indicates better optimization performance in the CEC2020 experiments, the detailed comparison results are summarized in Table 2, Table 3, Table 4, Table 5, Table 6, Table 7, Table 8, Table 9, Table 10 and Table 11.

From the perspective of average performance, EGRNOA achieves the best or tied-best Mean values on the ten CEC2020 functions. Specifically, EGRNOA obtains the tied-best result with the standard NOA on F1 and achieves better Mean values on F2–F10. The most obvious Mean advantages appear on F5 and F7, where the differences between EGRNOA and the second-best algorithm are 39.2771 and 36.3473, respectively. In terms of repeated-run stability, EGRNOA obtains the lowest Std values on six functions, namely, F1, F2, F4, F6, F7, and F8. These results indicate that the proposed search enhancement strategies can improve the competitiveness and stability of EGRNOA under the same function evaluation budget, while the difference between EGRNOA and the standard NOA still needs to be further judged through subsequent statistical tests.

To further investigate the statistical significance of the performance differences among the algorithms, this paper conducts statistical comparisons on the ten CEC2020 benchmark functions using the Friedman ranking test and Nemenyi post hoc analysis. The average ranking results are shown in Figure 3. EGRNOA achieves the best average ranking of 1.050, followed by the standard NOA with an average ranking of 1.950. The critical difference calculated at the 0.05 significance level is CD=2.8490. Since the ranking difference between EGRNOA and the standard NOA is smaller than the CD value, no statistically significant difference is observed between them; nevertheless, EGRNOA still obtains better average rankings than PSO, HPSO-LS-FS, ACO, GA, and HGAFS, and shows significant differences from several of these algorithms. These results indicate that EGRNOA has strong competitiveness and stability on the CEC2020 benchmark functions, verifying the global optimization capability of the EGRNOA search mechanism and providing a foundation for its application to discrete feature subspace search.

### 4.3. Comparison of EGRNOA with Other Metaheuristic Feature Selection Methods

Table 12, Table 13, Table 14, Table 15, Table 16, Table 17, Table 18, Table 19, Table 20 and Table 21 compare the results of EGRNOA with PSO, ACO, GA, the standard NOA, HGAFS, and HPSO-LS-FS on ten classification datasets. EGRNOA achieves the highest average accuracy on nine of the ten datasets, indicating that the combination of mRMR-based preselection, Logistic–Tent initialization, memory-differential spiral search, and elite-guided reconstruction can improve the quality of feature subsets across datasets with different feature dimensionalities.

Among the datasets on which EGRNOA ranks first, the largest advantage in average accuracy is observed on the movement dataset, where EGRNOA outperforms the second-best algorithm (HPSO-LS-FS) by 6.41 percentage points. In terms of absolute accuracy, movement (77.20%) and arcene (85.35%) remain relatively challenging datasets. On the arcene dataset, HPSO-LS-FS achieves the highest average accuracy of 86.06%, while EGRNOA obtains an average accuracy of 85.35%, which still represents a competitive performance.

Further considering the feature selection rate and running time, it can be observed that EGRNOA is able to maintain a reasonable feature subset size while improving classification accuracy. Compared with algorithms that search directly in the original feature space, mRMR-based preselection helps to reduce the interference of redundant and irrelevant features in the subsequent search process, enabling the algorithm to optimize feature subsets in a more compact candidate space. From the perspective of running time, EGRNOA’s introduction of Logistic–Tent initialization, memory-differential spiral search, and elite-guided reconstruction mechanisms results in higher computational overhead than some simple baseline algorithms; however, the overall cost remains within an acceptable range. Considering classification accuracy, feature selection rate, and running time together, EGRNOA achieves a good trade-off among classification performance, feature compression capability, and computational cost.

Figure 4 presents the convergence curves of the compared algorithms on the ten classification datasets. Unlike the statistical results in Table 12, Table 13, Table 14, Table 15, Table 16, Table 17, Table 18, Table 19, Table 20 and Table 21, which only reflect the final classification accuracy, the convergence curves further illustrate the search dynamics of each algorithm during the iterative process, including the speed at which the algorithm enters a higher-accuracy region, whether the curve remains stable in the later stage, and whether long-term oscillation or premature stagnation occurs. Therefore, this figure can provide additional evidence for the search stability of EGRNOA on datasets with different dimensionalities from the perspective of the optimization process.

Overall, EGRNOA exhibits a more favorable convergence trend on most datasets. On datasets such as lymphography, segmentation, sonar, and movement, EGRNOA usually reaches a higher accuracy level relatively quickly in the early iterations and maintains a comparatively stable curve shape in subsequent iterations. This indicates that Logistic–Tent initialization helps to improve the initial population coverage, while the diversity-adaptive elite-guided reconstruction mechanism can reduce ineffective oscillations after promising feature subsets are obtained. On datasets with greater search pressure, such as isolet5, cnae, rejafada, and arcene, some comparison algorithms show long-term fluctuations or stagnate early at relatively low accuracy levels, whereas EGRNOA can still continue to improve candidate feature subsets in the later iterations. Although HPSO-LS-FS remains highly competitive on the arcene dataset, the overall convergence process across the ten datasets shows that EGRNOA achieves a good balance between early-stage exploration speed and later-stage exploitation stability, consistent with the average accuracy results reported in Table 12, Table 13, Table 14, Table 15, Table 16, Table 17, Table 18, Table 19, Table 20 and Table 21.

Figure 5 further presents the distribution of classification accuracy for each algorithm over 20 independent runs. Unlike the mean statistics reported in Table 12, Table 13, Table 14, Table 15, Table 16, Table 17, Table 18, Table 19, Table 20 and Table 21, box plots can simultaneously display the median, interquartile range, extreme values, and possible abnormal fluctuations, making them more suitable for observing the robustness of algorithms under random initialization and stochastic search processes. In general, a higher box position indicates better typical classification performance over repeated runs, whereas a shorter box and more concentrated whiskers indicate that the algorithm is less sensitive to random perturbations and has stronger repeated-run stability.

From the overall distribution, the box positions of EGRNOA are at relatively high levels on most datasets, including lymphography, segmentation, sonar, movement, isolet5, parkinson, cnae, interad, and rejafada. This is consistent with the average accuracy comparison results in Table 12, Table 13, Table 14, Table 15, Table 16, Table 17, Table 18, Table 19, Table 20 and Table 21, indicating that the advantage of EGRNOA is not derived from a few occasional optimal results but from relatively stable classification performance across multiple independent runs. On several datasets, the boxes and whiskers of EGRNOA are relatively concentrated, indicating that the combination of mRMR-based preselection, Logistic–Tent initialization, memory-differential spiral search, and elite-guided reconstruction not only helps to improve the quality of feature subsets but also reduces performance fluctuations across repeated runs. On the arcene dataset, HPSO-LS-FS achieves the highest average accuracy; although EGRNOA is slightly lower, it still maintains a competitive level without showing obvious instability or dispersion. Therefore, the box plot results further support that EGRNOA provides stable and competitive feature selection capability across datasets with different dimensionalities.

Figure 6 presents the critical difference (CD) diagram obtained from the Nemenyi post hoc test on the ten classification datasets. This figure evaluates the overall performance of each algorithm based on the average ranking across multiple datasets, where a smaller average ranking indicates better overall performance. EGRNOA achieves the best average ranking of 1.100. At the 0.05 significance level, the critical difference threshold is CD=2.8490. The average ranking differences between EGRNOA and HGAFS, ACO, NOA, and PSO are 2.900, 3.600, 4.600, and 4.700, respectively, all of which exceed the CD threshold. This indicates that EGRNOA has a statistically significant overall advantage over these algorithms. The average ranking differences between EGRNOA and HPSO-LS-FS and GA are 2.200 and 2.300, respectively, which do not exceed the CD threshold, indicating that the differences between these two algorithms and EGRNOA do not reach statistical significance under the Nemenyi test. Nevertheless, EGRNOA still ranks first in terms of average ranking; when combined with the results in Table 12, Table 13, Table 14, Table 15, Table 16, Table 17, Table 18, Table 19, Table 20 and Table 21, where it achieves the highest average accuracy on nine out of the ten datasets, this further demonstrates that the proposed framework provides strong overall competitiveness in classification-oriented feature selection tasks.

### 4.4. Ablation Experiment

To further verify the actual contribution of each improved strategy in EGRNOA to the final performance, this paper conducts ablation experiments on five representative datasets: isolet5, parkinson, cnae, rejafada, and arcene. These datasets differ in feature scale and classification difficulty, making it possible to examine whether each module provides stable contributions under different data conditions rather than producing occasional effects on a single dataset.

The complete EGRNOA is compared with four reduced variants, as shown in Table 22. Specifically, EGRNOA w/o mRMR removes the mRMR-based relevance–redundancy preselection strategy; EGRNOA w/o LT removes Logistic–Tent chaotic initialization; EGRNOA w/o MDS removes the memory differential-guided spiral search strategy; and EGRNOA w/o EGR removes the diversity-adaptive elite-guided reconstruction mechanism for inferior individuals. All ablation variants adopt the same evaluation procedure as the preceding comparative experiments, so the performance differences mainly reflect the influence of the removed modules on the search capability of the algorithm.

Table 23 presents the classification accuracy (%) of the complete EGRNOA and its ablation variants on the five selected datasets, where a higher value indicates better classification performance after feature selection.

As shown in Table 23, the complete EGRNOA achieves the highest accuracy on all five datasets, indicating that the improved modules work synergistically rather than relying on the contribution of only a single operator. After removing mRMR, the accuracy results on isolet5, Parkinson, CNAE, rejafada, and arcene decrease by 2.90, 7.45, 4.51, 7.33, and 2.05 percentage points, respectively. The most obvious declines occur on Parkinson and rejafada, indicating that when the data contain many weakly relevant or redundant features, the prior relevance–redundancy screening can effectively reduce the burden of subsequent search.

The other ablation variants also lead to performance degradation to different extents. After removing Logistic–Tent initialization, the accuracy on each dataset decreases by 2.20–3.45 percentage points, indicating that a more uniformly distributed initial population helps to improve the quality of the search starting points. After removing memory-differential spiral search, the accuracy decreases by 2.25–3.25 percentage points, showing that the spiral search behavior integrating individual historical memory and random differential directions helps to further refine candidate feature subsets. After removing elite-guided reconstruction, the decreases on Parkinson, CNAE, and rejafada reach 5.44, 5.66, and 5.20 percentage points, respectively, indicating that reconstructing inferior individuals using elite information can prevent the population from remaining in low-quality regions for a long time. Figure 7 visually compares their relative contributions across the five benchmark datasets.

It can be further observed from Figure 7 that the bar height of the complete EGRNOA is higher than those of all reduced variants on all datasets, which is consistent with the numerical results in Table 23. Among them, removing mRMR and removing elite-guided reconstruction generally lead to more obvious performance degradation, indicating that data-level preselection and population-level reconstruction of inferior individuals are key components for maintaining algorithm performance. Therefore, the ablation experiments jointly demonstrate the necessity of the combined design of mRMR-based preselection, chaotic initialization, memory-differential spiral search, and elite-guided reconstruction from both numerical and visual perspectives.

To further distinguish the contributions of the mRMR-based preselection strategy and the subsequent search mechanism improvements to the final performance, this paper adds a controlled comparison experiment of NOA + mRMR under the same experimental settings. NOA + mRMR and EGRNOA use the same mRMR-based candidate feature space, but the search updating process of NOA + mRMR still follows the standard NOA without introducing Logistic–Tent chaotic initialization, memory-differential spiral search, or the elite-guided reconstruction mechanism for disadvantaged individuals. By comparing the standard NOA, NOA + mRMR, and EGRNOA, the effects of mRMR-based candidate space compression and the improved search mechanisms on classification accuracy can be observed separately.

As shown in Table 24, NOA + mRMR achieves higher average classification accuracy than the standard NOA on all six representative datasets, indicating that mRMR-based preselection can reduce the interference of redundant and irrelevant features in the search process of the standard NOA, thereby improving its feature selection performance. On this basis, EGRNOA further achieves higher average classification accuracy than NOA + mRMR on all six datasets, demonstrating that Logistic–Tent chaotic initialization, memory-differential spiral search, and elite-guided reconstruction of disadvantaged individuals can further improve the quality of feature subset search under the same mRMR-based candidate feature space. Therefore, the controlled comparison results indicate that the performance improvement of EGRNOA is not derived solely from mRMR-based preselection but from the joint effect of mRMR-based candidate space compression and the improved search mechanisms.

## 5. Conclusions

This paper proposes an Elite-Guided Nutcracker Optimization Algorithm with Memory-Differential Spiral Search for Feature Selection (EGRNOA). The proposed method integrates mRMR-based preselection, Logistic–Tent chaotic initialization, memory-differential spiral search, diversity-adaptive elite-guided reconstruction, reflective boundary handling, a probabilistic acceptance mechanism, and a sigmoid-based binary mapping strategy to improve the balance between exploration and exploitation during the search process and reduce the interference of redundant features in feature subset search. Experimental results on the CEC2020 benchmark functions show that EGRNOA achieves the best or tied-best average performance on all ten test functions and obtains the best Friedman average ranking. Under the current evaluation protocol, EGRNOA achieves the highest average classification accuracy on nine out of ten classification datasets and remains highly competitive on the remaining dataset. The convergence curves, box plots, CD diagram, and ablation experiments further support the effectiveness of the proposed improvement modules in enhancing search stability, robustness across repeated runs, and classification performance.

Although EGRNOA demonstrates strong overall competitiveness, this study still has several limitations. First, mRMR uses class label information and performs feature ranking on the complete dataset prior to five-fold cross-validation; therefore, label information from the validation folds may indirectly influence the determination of the candidate feature set. In addition, this study uses five-fold cross-validation accuracy to guide feature subset search and reports the final classification performance based on the same cross-validation results, which may introduce a certain degree of optimistic bias into the accuracy estimates. Therefore, the classification accuracy results reported in this study are primarily intended to compare the relative performance of different algorithms under the current unified evaluation protocol, and should not be interpreted as fully independent estimates of out-of-sample generalization performance. Second, this study mainly uses the KNN classifier to evaluate candidate feature subsets; the applicability of the selected features to other classification models such as SVM, random forests, and neural networks has not been further validated. Finally, although the selected datasets cover different dimensional scales, real-world biomedical data can involve more complex issues such as class imbalance, missing values, noise interference, and small sample sizes. Future work will employ nested cross-validation or independent test sets with feature preselection and feature subset search performed entirely within the training data, and will further evaluate the generalization performance, scalability, and practical applicability of EGRNOA using different classifiers and more real-world biomedical datasets.

## Figures and Tables

**Figure 1:**
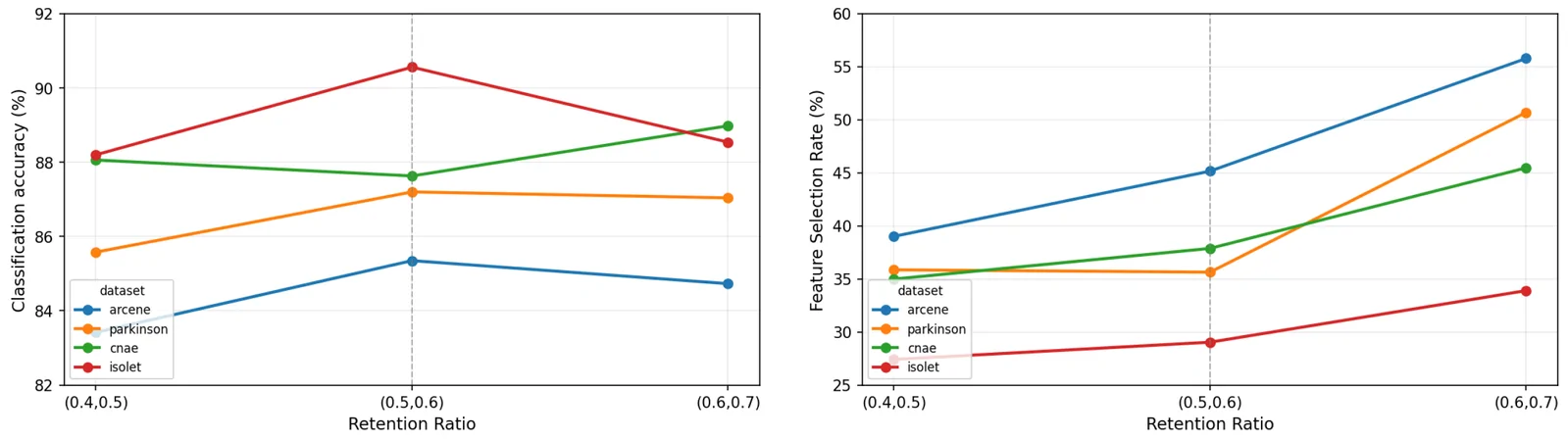
mRMR feature retention ratio.

**Figure 2:**
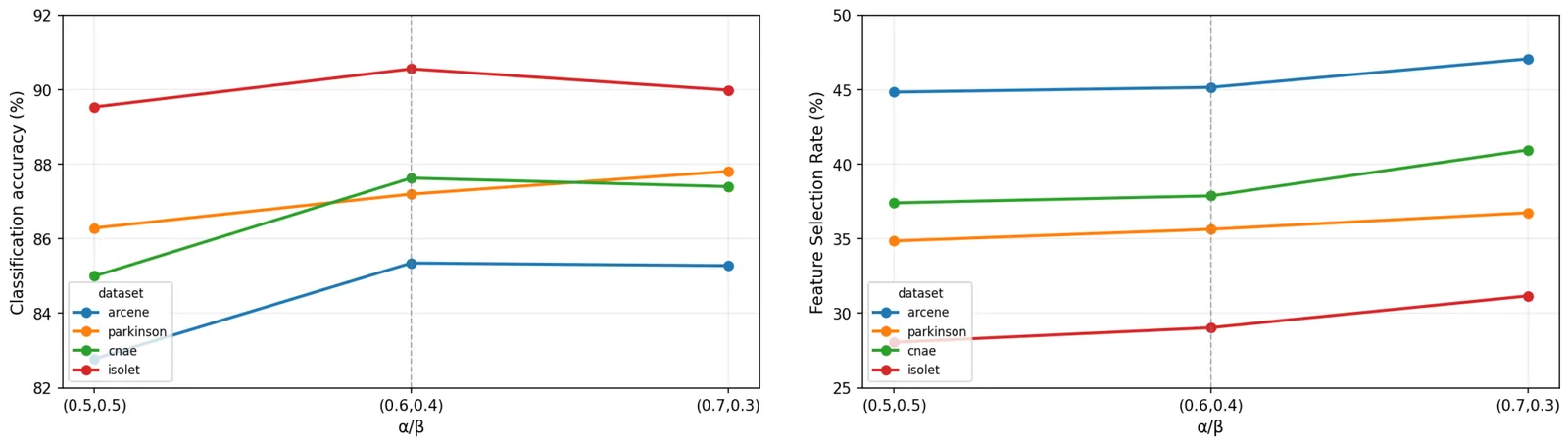
Fitness function weights.

**Figure 3:**
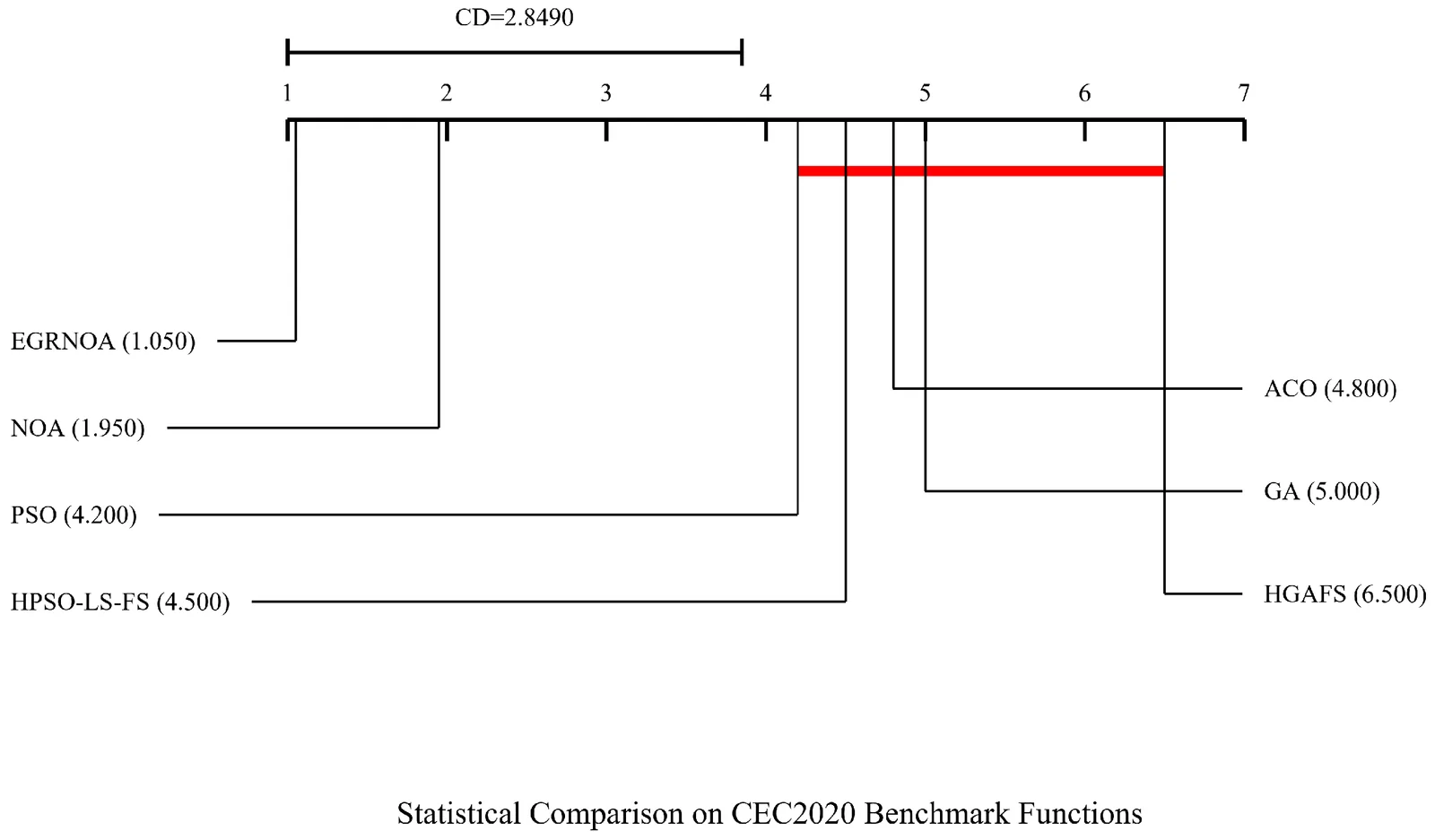
CD diagram of the compared algorithms on the CEC2020 benchmark functions.

**Figure 4:**
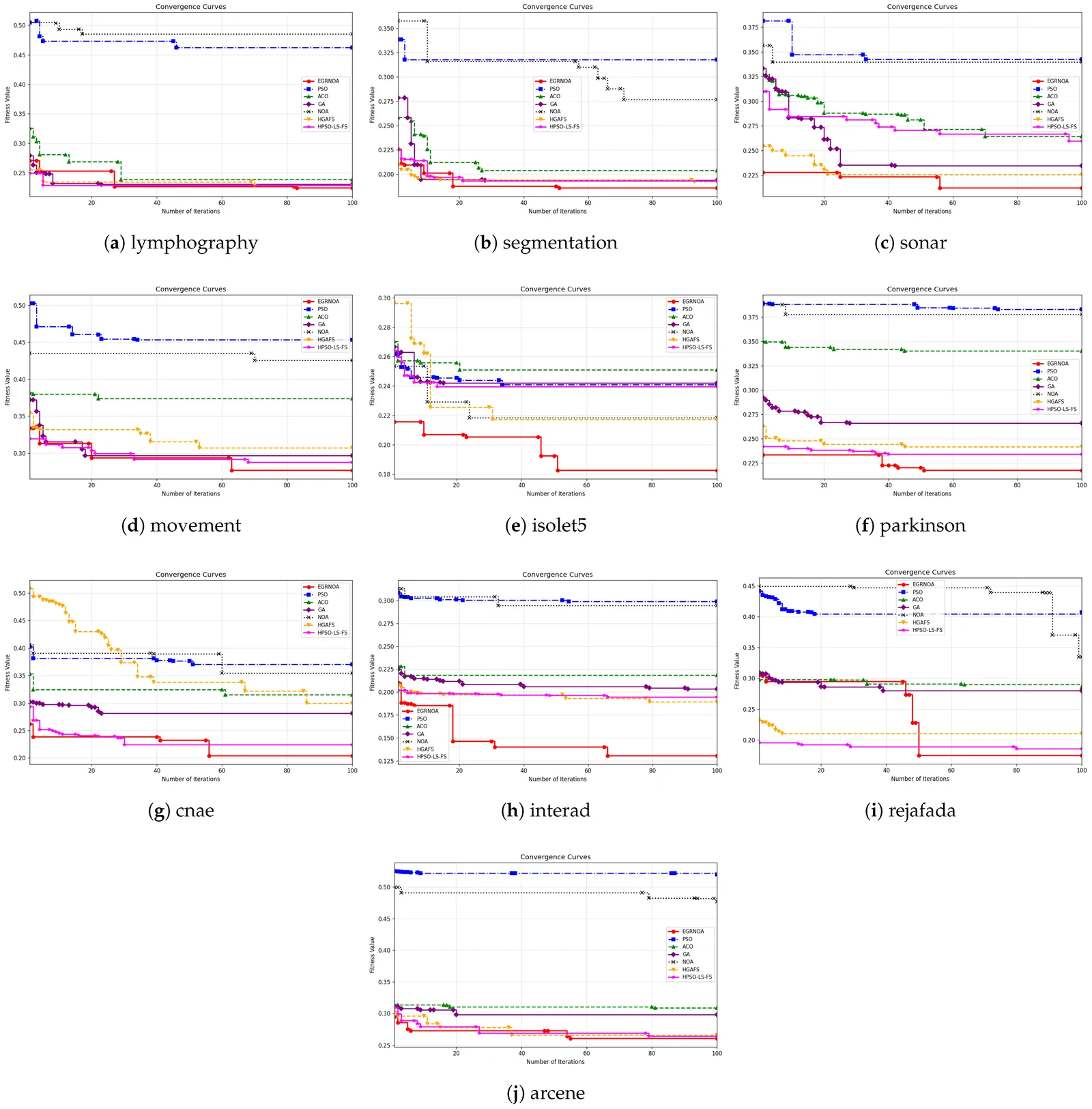
Convergence curves of the compared algorithms on the ten classification datasets.

**Figure 5:**
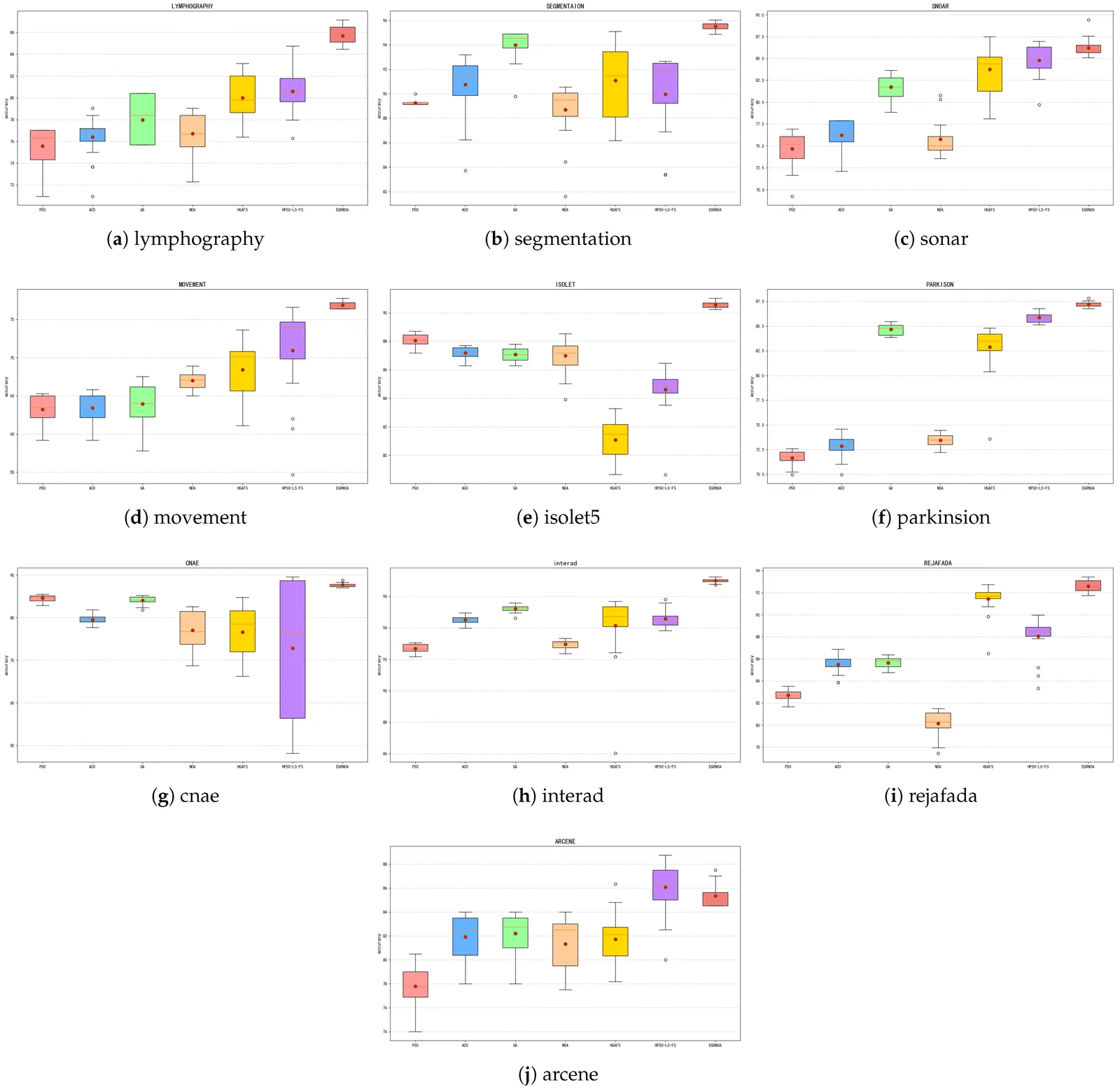
Box plot of classification accuracy on all datasets.

**Figure 6:**
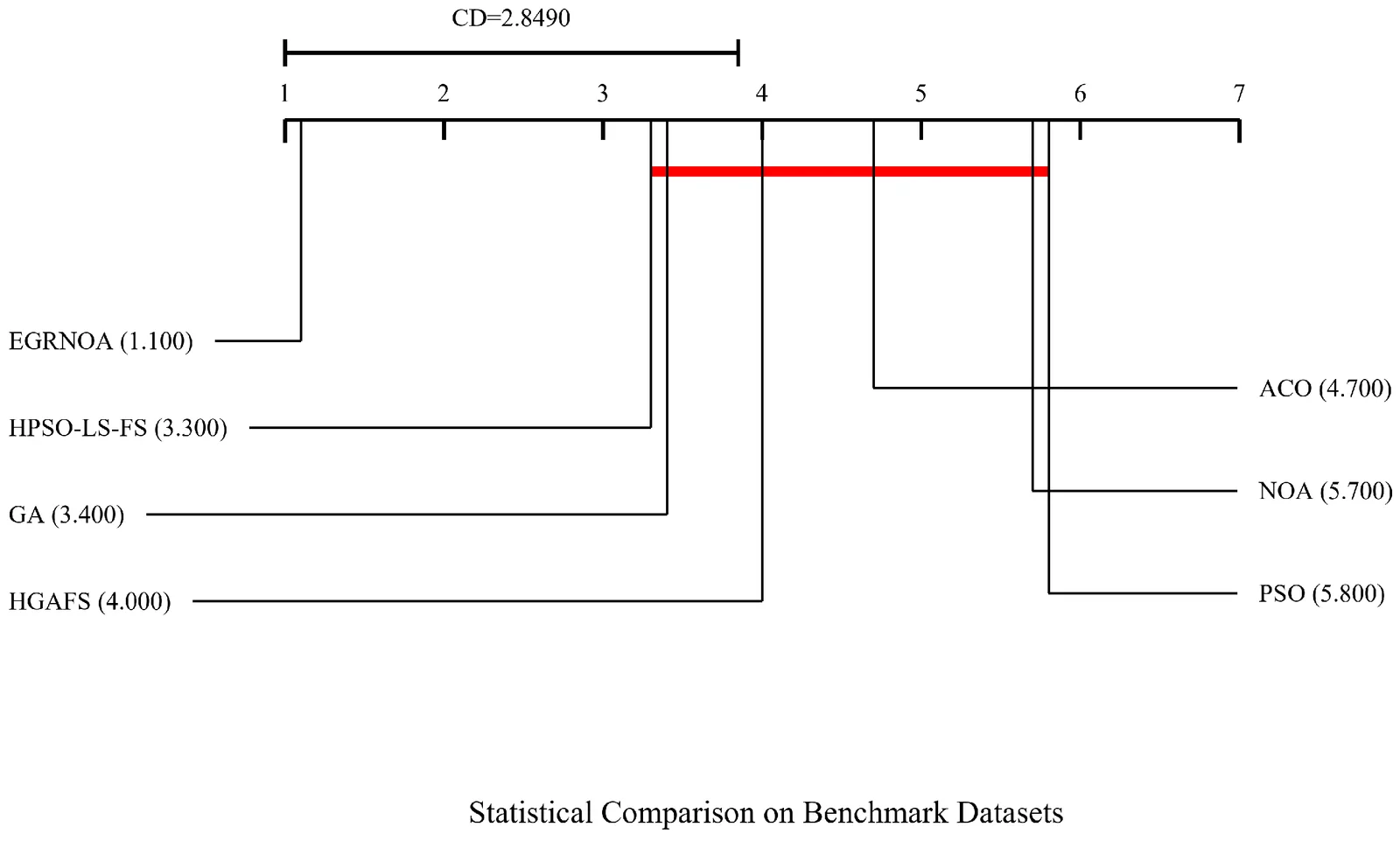
CD diagram of the compared algorithms on all datasets.

**Figure 7:**
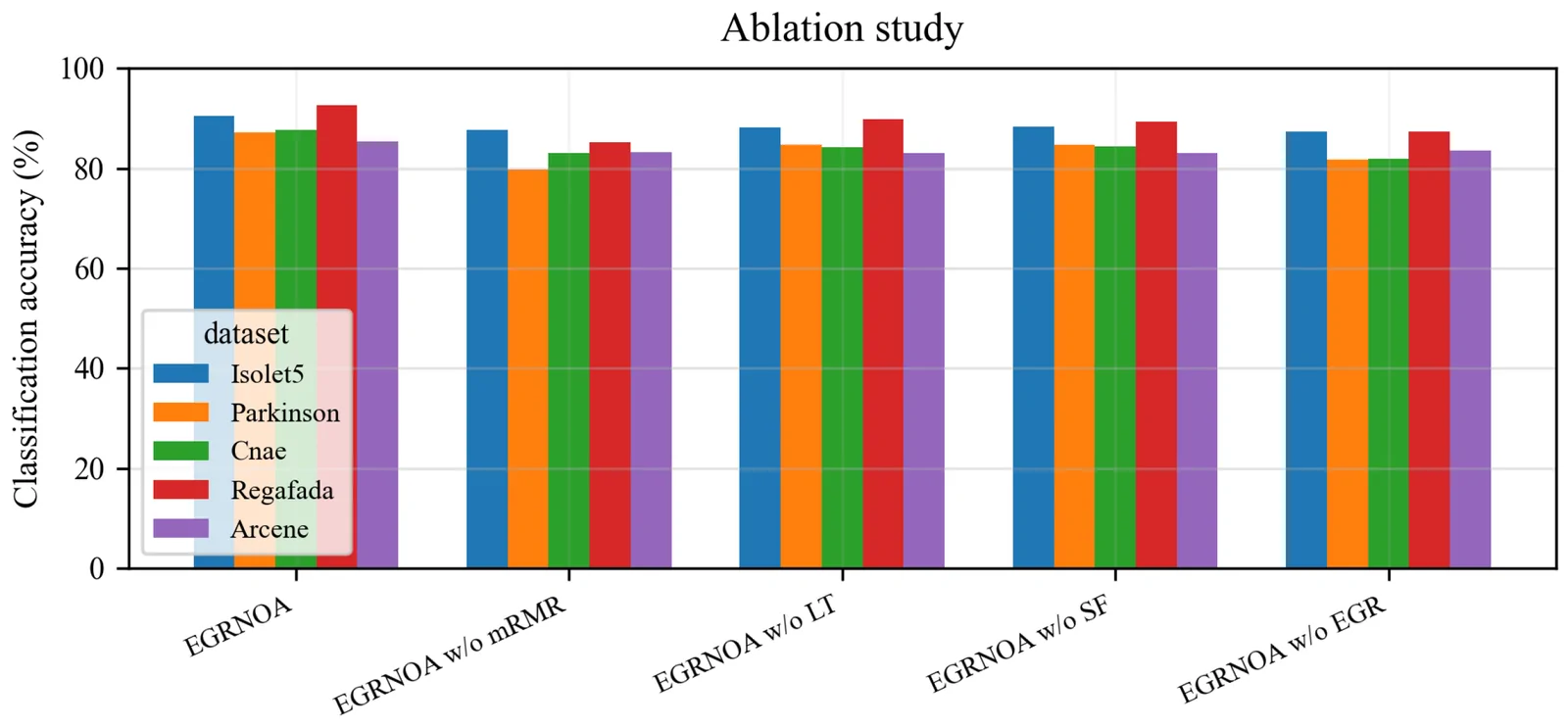
Comparison of EGRNOA and its ablation variants on the selected datasets.

**Table 1:** Summary of classification benchmark dataset information.

No.	Dataset	Samples	Features	Classes
1	lymphography	148	18	4
2	segmentation	2310	19	7
3	sonar	208	60	2
4	movement	360	90	15
5	isolet5	1559	617	26
6	Parkinson’s Disease Classification	756	754	2
7	cnae	1080	856	9
8	Internet Advertisements	3279	1588	2
9	rejafada	1996	6824	2
10	arcene	200	10,000	2

**Table 2:** Experimental results on CEC2020 benchmark function F1.

Algorithm	Best	Worst	Mean	Std
EGRNOA	**100**	**100**	**100**	**0**
ACO	211.8533	5193.79	1606.15	1311.67
PSO	711.7479	8232.44	3467.30	2485.85
GA	1709.24	12,385.00	7241.70	3848.84
NOA	**100**	**100**	**100**	**0**
HGAFS	4,485,139.24	8,553,585.02	6,201,084.04	1,264,016.87
HPSO-LS-FS	1141.87	7805.47	3218.40	1848.17

**Table 3:** Experimental results on CEC2020 benchmark function F2.

Algorithm	Best	Worst	Mean	Std
EGRNOA	**1101.89**	**1246.02**	**1165.41**	**59.3296**
ACO	2152.74	3718.91	2581.03	430.6335
PSO	2553.69	3763.22	2944.32	354.1770
GA	1611.14	2310.42	1916.35	226.0713
NOA	1102.08	1328.71	1192.05	67.0247
HGAFS	2481.56	3563.42	2992.64	374.1841
HPSO-LS-FS	1469.09	1921.30	1651.67	123.7497

**Table 4:** Experimental results on CEC2020 benchmark function F3.

Algorithm	Best	Worst	Mean	Std
EGRNOA	**722.4656**	**732.9414**	**727.6204**	2.7918
ACO	737.7863	758.6807	744.5485	4.7413
PSO	763.7781	839.1095	788.6017	20.2968
GA	729.2772	748.6704	735.2549	5.4993
NOA	724.9840	735.3075	730.3961	2.9946
HGAFS	791.5584	840.3821	806.8706	15.1485
HPSO-LS-FS	778.9067	785.1779	781.3664	**1.9826**

**Table 5:** Experimental results on CEC2020 benchmark function F4.

Algorithm	Best	Worst	Mean	Std
EGRNOA	**1900.72**	**1901.53**	**1901.29**	**0.1993**
ACO	1902.75	1915.00	1905.09	2.8352
PSO	1903.21	1909.00	1904.93	1.3553
GA	1902.43	1906.06	1903.61	1.0874
NOA	1901.14	1902.32	1901.69	0.3572
HGAFS	1904.09	1908.37	1905.08	1.1048
HPSO-LS-FS	1904.77	1906.85	1905.74	0.6512

**Table 6:** Experimental results on CEC2020 benchmark function F5.

Algorithm	Best	Worst	Mean	Std
EGRNOA	**1730.87**	**1970.36**	**1850.74**	73.8660
ACO	21,880.28	171,009.66	59,714.93	34,323.48
PSO	18,278.36	132,248.39	51,825.37	26,556.62
GA	46,630.74	2,676,085.57	104,960.77	611,816.36
NOA	1841.94	2020.45	1890.02	**48.8689**
HGAFS	87,643.29	176,272.50	113,596.48523	25,248.64217
HPSO-LS-FS	25,318.64544	132,248.39304	57,551.91057	26,776.48924

**Table 7:** Experimental results on CEC2020 benchmark function F6.

Algorithm	Best	Worst	Mean	Std
EGRNOA	**1600.32**	**1601.88**	**1601.20**	**0.5353**
ACO	1726.37	2057.53	1848.66	100.4663
PSO	1846.41	2266.37	1966.39	99.8402
GA	1726.65	1962.50	1858.17	92.0735
NOA	1600.88	1602.59	1601.44	0.57054
HGAFS	1953.74	2268.60	2028.43	84.3993
HPSO-LS-FS	1601.62	1721.57	1607.91	26.0754

**Table 8:** Experimental results on CEC2020 benchmark function F7.

Algorithm	Best	Worst	Mean	Std
EGRNOA	**2102.08**	**2146.12**	**2123.67**	**13.0182**
ACO	2768.21	32,277.43	8528.31	8221.29
PSO	5869.89	22,414.22	10,581.44	5547.70
GA	280,411.44	1,264,214.67	558,662.60	287,576.99
NOA	2111.88	2230.46	2160.02	43.2751
HGAFS	11,193.17	15,783.94	12,810.63	1262.44
HPSO-LS-FS	22,843.95	151,014.56	58,177.63	30,354.39

**Table 9:** Experimental results on CEC2020 benchmark function F8.

Algorithm	Best	Worst	Mean	Std
EGRNOA	**2300**	**2300**	**2300**	**0**
ACO	2300.00	2304.98	2301.67	1.20835
PSO	2300	5859.37	4126.83	1302.98
GA	2301.43	5527.22	3885.99	1216.02
NOA	**2300**	2301.33	2300.20	0.4513
HGAFS	5826.85	5860.23	5840.31	9.7316
HPSO-LS-FS	2301.28	4909.82	3067.68	1057.69

**Table 10:** Experimental results on CEC2020 benchmark function F9.

Algorithm	Best	Worst	Mean	Std
EGRNOA	**2806.69**	**2824.60**	**2817.53**	4.5321
ACO	2825.67	2857.77	2838.85	7.8638
PSO	2869.58	2971.94	2895.98	26.1454
GA	2909.11	3077.61	2960.31	53.0251
NOA	2815.97	2833.96	2825.35	6.6949
HGAFS	3002.44	3005.00	3003.59	**0.7805**
HPSO-LS-FS	2839.84	2892.55	2857.28	13.5536

**Table 11:** Experimental results on CEC2020 benchmark function F10.

Algorithm	Best	Worst	Mean	Std
EGRNOA	**2913.85**	**2960.22**	**2924.88**	17.7923
ACO	2977.24	3007.40	2996.52	7.7805
PSO	2914.13	3005.58	2968.80	27.8358
GA	2948.13	3004.14	2982.29	17.2185
NOA	2913.75	2993.83	2936.06	31.0717
HGAFS	3004.40	3007.11	3005.72	**0.8684**
HPSO-LS-FS	2969.56	3009.48	2989.25	12.4493

Note: Bold font indicates the optimal value in the comparative data.

**Table 12:** Experimental results on the lymphography dataset.

Algorithm	Best	Worst	Mean	Std	Feature Selection Rate (%)	Time
EGRNOA	**0.8716**	**0.8446**	**0.8571**	**0.0075**	37.56	2.85
PSO	0.7838	0.7027	0.7605	0.0181	72.22	2.53
ACO	0.7905	0.7095	0.7639	0.0189	**22.96**	7.30
GA	0.8041	0.7297	0.7885	0.0204	31.11	6.06
NOA	0.7905	0.7230	0.7672	0.0193	83.15	3.21
HGAFS	0.8315	0.7640	0.7961	0.0273	40.00	6.23
HPSO-LS-FS	0.8475	0.7627	0.8059	0.0211	33.33	**2.50**

**Table 13:** Experimental results on the segmentation dataset.

Algorithm	Best	Worst	Mean	Std	Feature Selection Rate (%)	Time
EGRNOA	**0.9605**	**0.9488**	**0.9550**	0.0031	43.68	8.66
PSO	0.9000	0.8913	0.8926	**0.0019**	65.44	8.65
ACO	0.9320	0.8372	0.9075	0.0235	42.00	28.40
GA	0.9489	0.8978	0.9399	0.0129	41.47	18.01
NOA	0.9055	0.8161	0.8869	0.0218	57.02	**7.05**
HGAFS	0.9510	0.8617	0.9167	0.0269	43.86	303
HPSO-LS-FS	0.9267	0.8338	0.8996	0.0353	**38.42**	7.27

**Table 14:** Experimental results on the sonar dataset.

Algorithm	Best	Worst	Mean	Std	Feature Selection Rate (%)	Time
EGRNOA	**0.8941**	**0.8511**	**0.8622**	**0.0097**	43.3	10.89
PSO	0.7692	0.6923	0.7466	0.0194	44.72	42.34
ACO	0.7788	0.7212	0.7622	0.0167	33.39	24.26
GA	0.8365	0.7885	0.8173	0.0142	**38.33**	126.43
NOA	0.8077	0.7356	0.7577	0.0185	50.61	**9.66**
HGAFS	0.8750	0.7812	0.8375	0.0306	34.00	10.1
HPSO-LS-FS	0.8696	0.7971	0.8478	0.0177	43.37	20.4

**Table 15:** Experimental results on the movement dataset.

Algorithm	Best	Worst	Mean	Std	Feature Selection Rate (%)	Time
EGRNOA	**0.7806**	**0.7667**	**0.7720**	**0.0048**	34.78	10.28
PSO	0.6528	0.5917	0.6321	0.0168	53.04	16.87
ACO	0.6583	0.5917	0.6342	0.0199	35.52	15.93
GA	0.6750	0.5778	0.6396	0.0237	**26.52**	17.9
NOA	0.6889	0.6500	0.6700	0.0103	57.85	**5.39**
HGAFS	0.7361	0.6111	0.6840	0.0407	33.63	25.13
HPSO-LS-FS	0.7667	0.5467	0.7079	0.0509	38.89	25.55

**Table 16:** Experimental results on the isolet5 dataset.

Algorithm	Best	Worst	Mean	Std	Feature Selection Rate (%)	Time
EGRNOA	**0.9102**	**0.9024**	**0.9056**	**0.0022**	**29.04**	54.34
PSO	0.8811	0.8660	0.8775	0.0032	42.90	746.12
ACO	0.8770	0.8628	0.8717	0.0040	45.11	1148.27
GA	0.8778	0.8629	0.8708	0.0046	40.99	1244.03
NOA	0.8697	0.8290	0.8569	0.0114	41.69	**30.02**
HGAFS	0.8327	0.7865	0.8107	0.0133	32.4	1228.29
HPSO-LS-FS	0.8647	0.7864	0.8406	0.0190	48.4	364.1

**Table 17:** Experimental results on the Parkinson’s Disease Classification dataset.

Algorithm	Best	Worst	Mean	Std	Feature Selection Rate (%)	Time
EGRNOA	**0.8783**	**0.8677**	**0.8720**	**0.0025**	**35.64**	15.37
PSO	0.7262	0.6997	0.7167	0.0074	54.23	41.15
ACO	0.7460	0.6997	0.7284	0.0105	45.31	37
GA	0.8545	0.8386	0.8466	0.0056	42.17	44.56
NOA	0.7447	0.7222	0.7345	0.0062	55.59	**9.72**
HGAFS	0.8480	0.7357	0.8287	0.0242	39.88	24.62
HPSO-LS-FS	0.8680	0.8515	0.8588	0.0053	41.53	14.11

**Table 18:** Experimental results on the cnae dataset.

Algorithm	Best	Worst	Mean	Std	Feature Selection Rate (%)	Time
EGRNOA	0.8870	**0.8704**	**0.8763**	**0.0046**	**37.88**	**10.82**
PSO	0.8546	0.8287	0.8458	0.0081	69.05	69.69
ACO	0.8185	0.7769	0.7952	0.0104	48.83	54.21
GA	0.8519	0.8176	0.8405	0.0095	46.70	73.81
NOA	0.8176	0.6870	0.7600	0.0326	63.52	10.9
HGAFS	0.8472	0.7269	0.7831	0.0481	48.26	623.7
HPSO-LS-FS	**0.8958**	0.4815	0.7018	0.1532	39.79	35.52

**Table 19:** Experimental results on the Internet Advertisements dataset.

Algorithm	Best	Worst	Mean	Std	Feature Selection Rate (%)	Time
EGRNOA	**0.9725**	**0.9674**	**0.9700**	**0.0013**	**27.4**	283.95
PSO	0.9305	0.9216	0.9269	0.0027	63.82	934.81
ACO	0.9496	0.9398	0.9452	0.0023	46.53	621.06
GA	0.9559	0.9462	0.9522	0.0021	43.76	722.25
NOA	0.9334	0.9237	0.9296	0.0024	61.67	**212.58**
HGAFS	0.9568	0.8603	0.9415	0.0209	32.74	277.36
HPSO-LS-FS	0.9581	0.9383	0.9458	0.0060	43.7	246.39

**Table 20:** Experimental results on the rejafada dataset.

Algorithm	Best	Worst	Mean	Std	Feature Selection Rate (%)	Time
EGRNOA	**0.9344**	**0.9173**	**0.9258**	0.0054	32.43	62.5
PSO	0.8342	0.8166	0.8262	0.0053	47.14	1378.76
ACO	0.8687	0.8387	0.8549	0.0080	49.77	1449.27
GA	0.8637	0.8477	0.8564	**0.0046**	49.34	1748.8
NOA	0.8151	0.7745	0.8014	0.0113	56.58	**55.29**
HGAFS	0.9274	0.8648	0.9124	0.0150	42.79	323.12
HPSO-LS-FS	0.8997	0.8333	0.8808	0.0167	**32.27**	361.9

**Table 21:** Experimental results on the arcene dataset.

Algorithm	Best	Worst	Mean	Std	Feature Selection Rate (%)	Time
EGRNOA	0.8750	**0.8450**	0.8535	**0.0085**	**45.16**	83.09
PSO	0.8050	0.7400	0.7778	0.0167	58.86	779.75
ACO	0.8400	0.7800	0.8192	0.0191	49.49	93.49
GA	0.8400	0.7800	0.8220	0.0160	49.35	104.01
NOA	0.8400	0.7750	0.8133	0.0216	86.52	68.7
HGAFS	0.8633	0.7817	0.8171	0.0220	48.12	1907.19
HPSO-LS-FS	**0.8875**	0.8000	**0.8606**	0.0217	50.47	**62.04**

Note: Bold font indicates the optimal value in the comparative data.

**Table 22:** Description of the ablation variants.

Variant	Description
EGRNOA	Complete method, including mRMR, Logistic–Tent initialization, memory-differential spiral search, and the diversity-adaptive elite-guided reconstruction mechanism
EGRNOA w/o mRMR	The mRMR-based preselection strategy is removed, and the search is directly performed in the original feature space
EGRNOA w/o LT	Logistic–Tent chaotic initialization is replaced with random initialization
EGRNOA w/o MDS	The memory-differential spiral search strategy in the foraging phase is removed
EGRNOA w/o EGR	The diversity-adaptive elite-guided reconstruction mechanism for inferior individuals is removed

**Table 23:** Results of EGRNOA and its ablation variants on selected datasets (accuracy, %).

Variant	isolet5	Parkinson	Cnae	Rejafada	Arcene
EGRNOA	**90.56**	**87.20**	**87.63**	**92.58**	**85.35**
EGRNOA w/o mRMR	87.66	79.75	83.12	85.25	83.30
EGRNOA w/o LT	88.21	84.79	84.18	89.79	83.15
EGRNOA w/o MDS	88.31	84.75	84.42	89.33	83.03
EGRNOA w/o EGR	87.31	81.76	81.97	87.38	83.58

**Table 24:** Comparison of average classification accuracy (%) among NOA, NOA + mRMR, and EGRNOA on representative datasets.

Dataset	NOA	NOA + mRMR	EGRNOA
Arcene	81.33	82.15	**85.35**
REJAFADA	80.14	84.59	**92.58**
interad	92.96	93.73	**97.00**
CNAE	76.00	80.37	**87.63**
parkinson	73.45	76.59	**87.20**
isolet	85.69	86.96	**90.56**

## Data Availability

All datasets used in this study were obtained from the open-access UCI Machine Learning Repository (https://archive.ics.uci.edu/, accessed on 15 January 2026).
